# Impact of the COVID-19 Pandemic on the Use and Outcomes of Cardiac Procedures in COPD Patients

**DOI:** 10.3390/jcm11133924

**Published:** 2022-07-05

**Authors:** Javier de Miguel-Diez, Rodrigo Jimenez-Garcia, Jose M. de Miguel-Yanes, Valentin Hernández-Barrera, David Carabantes-Alarcon, Jose J. Zamorano-Leon, Concepción Noriega, Ana Lopez-de-Andres

**Affiliations:** 1 Respiratory Care Department, Hospital General Universitario Gregorio Marañón, Instituto de Investigación Sanitaria Gregorio Marañón (IiSGM), Universidad Complutense de Madrid, 28007 Madrid, Spain; javier.miguel@salud.madrid.org; 2 Department of Public Health and Maternal & Child Health, Faculty of Medicine, IdISSC, Universidad Complutense de Madrid, 28040 Madrid, Spain; dcaraban@ucm.es (D.C.-A.); josejzam@ucm.es (J.J.Z.-L.); anailo04@ucm.es (A.L.-d.-A.); 3 Internal Medicine Department, Hospital General Universitario Gregorio Marañón, Instituto de Investigación Sanitaria Gregorio Marañón (IiSGM), Universidad Complutense de Madrid, 28007 Madrid, Spain; josemaria.demiguel@salud.madrid.org; 4 Preventive Medicine and Public Health Teaching and Research Unit, Health Sciences Faculty, Universidad Rey Juan Carlos, 28922 Alcorcón, Spain; valentin.hernandez@urjc.es; 5 Department of Nursery and Physiotherapy, Faculty of Medicine and Health Sciences, University of Alcalá, 28871 Alcalá de Henares, Spain; concha.noriega@uah.es

**Keywords:** COPD, COVID-19, cardiac procedures, hospitalization

## Abstract

(1) Background: The aim of this study was to assess the effects of the COVID-19 pandemic on the use and outcomes of cardiac procedures among people with chronic obstructive pulmonary disease (COPD) in Spain. (2) Methods: We used national hospital discharge data to select patients admitted to hospital with a diagnosis of COPD from 1 January 2019 to 31 December 2020. (3) Results: The number of COPD patients hospitalized in 2019 who underwent a cardiac procedure was 4483, 16.2% higher than in 2020 (n = 3757). The length of hospital stay was significantly lower in 2020 than in 2019 (9.37 vs. 10.13 days; *p* = 0.004), and crude in-hospital mortality (IHM) was significantly higher (5.32% vs. 4.33%; *p* = 0.035). Multivariable logistic regression models to assess the differences in IHM from 2019 to 2020 showed Odds Ratio (OR) values over 1, suggesting a higher risk of dying in 2020 compared to in 2019. However, the ORs were only statistically significant for “any cardiac procedure” (1.18, 95% CI 1.03–1.47). The Charlson comorbidity index increased IHM for each of the procedures analyzed. The probability of IHM was higher for women and older patients who underwent coronary artery bypass graft or open valve replacement procedures. Suffering a COVID-19 infection was associated with significantly higher mortality after cardiac procedures. (4) Conclusions: The COVID-19 pandemic limited the access to healthcare for patients with COPD.

## 1. Introduction

The COVID-19 pandemic has had a dramatic impact on healthcare systems. Many ambulatory visits and surgeries were initially canceled [1]. Consequently, it increased the risk of morbidity and mortality associated with several health conditions [2]. This impact was greater in vulnerable patients, such as those with chronic cardiopulmonary diseases [3].

About 75% of patients hospitalized with COVID-19 infection have at least one associated comorbidity [4]. Furthermore, chronic obstructive pulmonary disease (COPD) patients hospitalized for COVID-19 infection have significantly more comorbidities than those without COPD as well as a higher risk of severe outcome and death within 30 days. Comorbidities, especially cardiovascular diseases, are associated with mortality among COPD patients [5].

Prioritization among patients with heart diseases requiring intervention has represented a difficult issue during the COVID-19 pandemic. Guidelines initially suggested postponing elective cardiovascular surgery [6]. However, cases undergoing emergency interventions also fell during the different waves of the COVID-19 pandemic [7], which might reflect the tendency that patients were less likely to visit hospitals to avoid exposure to COVID-19 infection, as suggested by previous studies [8,9,10]. In any case, postponing cardiac procedures could have restricted the daily activities of these patients and could have increased the risk of cardiovascular complications.

Despite this, and to our knowledge, there are no data on cardiac procedures carried out during the pandemic in COPD patients with cardiovascular disorders, a particularly vulnerable subpopulation that is both at high risk for COVID-19 infection complications and from the negative consequences of delayed medical care [11]. Hospitals having reduced capacity to treat conditions other than COVID-19 infections coupled with staffing shortages and resource limitations [12] could have led to significant deviations from the established gold standards for cardiac procedures in these patients.

To address these issues, we aim to assess the effects of the COVID-19 pandemic on the use and outcomes of cardiac procedures among people with COPD in Spain.

## 2. Materials and Methods

We conducted a descriptive epidemiological study using the Spanish National Hospital Discharge Database (SNHDD). The SNHDD is an administrative database managed by the Spanish Ministry of Health (SMH) that collects information from all private and public hospitals. These hospitals are required by law to provide data from all subjects hospitalized for at least 24 h. The following variables for each patient are included in the SNHDD: age, sex, place of residence, dates of admission and discharge, discharge destination, primary diagnosis, secondary diagnosis (up to a maximum of 19), and procedures (therapeutic or diagnostic) conducted during the hospitalization period (up to a maximum of 20). To codify diagnoses and procedures, the SNHDD applies the International Classification of Disease Tenth Revision (ICD10). Details on the SNHDD can be found online [13]. The study period was from 1 January 2019 to 31 December 2020.

### 2.1. Study Population and Variables

The study population included all patients who were admitted and discharged during this time period and who received a code, in any diagnosis position, for COPD (ICD10 codes J44.0, J44.1, and J44.9). We excluded those aged under 40 years old and those with missing data for age, sex, duration of hospitalization, or discharge destination.

The cardiac procedures analyzed included percutaneous coronary intervention (PCI); coronary artery bypass graft (CABG); open valve replacement procedures (OVRP) for aortic, mitral, pulmonary, and tricuspid valves; and transcatheter valve replacement (TVR) for aortic, mitral, pulmonary, and tricuspid valves. The ICD10 codes for these procedures are shown in Table S1.

The main outcome variables were the number of these procedures conducted in 2019 compared to in 2020. Secondary outcomes were in-hospital mortality (IHM) and length of hospital stay (LOHS). IHM is the proportion of patients who had “deceased during hospitalization” as their discharge location.

The demographic and clinical characteristic analyzed were age, sex, Charlson comorbidity index (CCI), clinical conditions (diabetes, asthma, stroke, peripheral vascular disease, ischemic heart disease, valvular heart disease, atrial fibrillation, heart failure, chronic kidney disease, and infection with COVID-19), oxygen prior to hospital admission, procedures required during hospitalization (non-invasive mechanical ventilation, invasive mechanical ventilation, and dialysis), and admission to the intensive care unit (ICU). The CCI was calculated for each patient using the algorithms defined by Quan et al. [14].

### 2.2. Statistical Analysis

In addition to analyzing the effect of the COVID-19 pandemic on the use of each of the four procedures studied, we created a new global variable that included all four procedures. This variable was denoted as “any cardiac procedure”.

The study variables were described using total frequencies, means with standard deviation, and proportions. The changes in the use of procedures from 2019 to 2020 was expressed as a proportion obtained by subtracting to the number of procedures conducted in 2019 from those performed in 2020 and then first dividing the number before multiplying it by one hundred.

To compare demographic and clinical conditions between 2019 and 2020, we used Student’s *t*-test or the Wilcoxon–Mann–Whitney test to compare the means and the Chi Square test to compare the proportions.

To assess the possible effect of changes in the distribution by age, sex, and comorbidity on IHM from 2019 to 2020, we applied multivariable logistic regression models, generating one model for each procedure and another model for “any cardiac procedure”. The models were constructed following the recommendations of Hosmer et al. [15].

### 2.3. Sensitivity Analysis

To control possible fluctuations in the outcomes of the cardiac procedures carried out in the years before the COVID-19 pandemic, we also analyzed IHM for the years 2016, 2017, and 2018 using methods identical to those described for 2019 and 2020. The Cochran–Armitage test was applied to assess time trends in IHM.

To assess the effect of a COVID-19 infection on the hospital outcomes of patients who underwent a cardiac procedure in 2020 we have compared patients with and without this infection. Furthermore, we have also compared each patient with a COVID-19 infection to a matched patient without infection from 2019 who underwent the same procedure with identical age, sex, and month of intervention variables.

The statistical software used was Stata 14 (StataCorp. College Station, TX, USA).

### 2.4. Ethical Aspects

The SNHDD databases were provided to us by the SMH after completing an application that can be downloaded at the link in [16]. It is not possible to identify the subjects included in the database as it is completely anonymous. According to Spanish legislation, the use of administrative data provided by the health authorities does not require authorization from an ethics committee.

## 3. Results

There were 4483 and 3757 people hospitalized in 2019 and 2020, respectively, who were diagnosed with COPD aged 40 years or older who underwent a cardiac procedure. The overall proportion of men was 86.13%, and the mean age was 71.76 years (SD 8.45) (Table 1).

Over the entire study period, the most common procedures were PCI (n = 5852), OVRP (n = 1098), CABG (n = 848), and TVR (n = 745).

**Figure 1:**
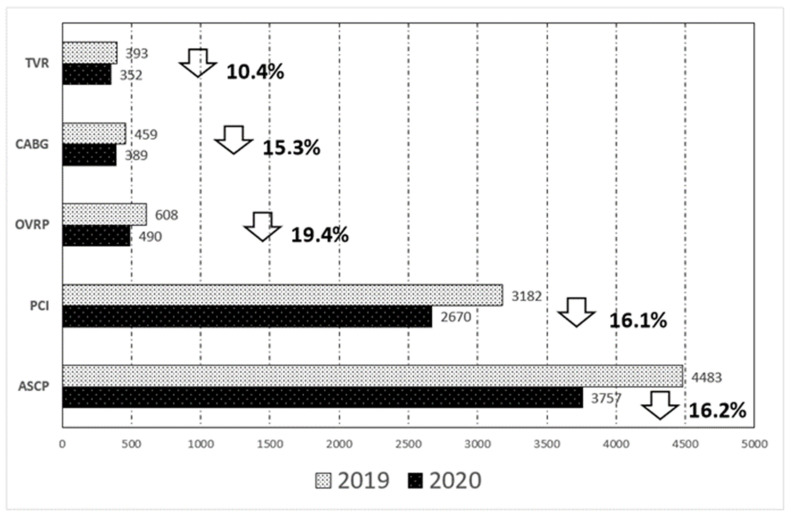
Number of cardiac procedures in patients with chronic obstructive pulmonary disease and the proportional change in “any cardiac procedure”, percutaneous coronary intervention, open valve replacement procedures, coronary artery bypass graft, and transcatheter valve replacement from 2019 to 2020. ASCP: Any cardiac procedure; PCI: Percutaneous Coronary Intervention; OVRP Open Valve Replacement Procedures. CABG: Coronary Artery Bypass Graft; TVR: Trans Catheter Valve Replacement.

The proportional reductions in “any cardiac procedure”, PCI, OVRP, CABG, and TVR from 2019 to 2020 are shown in Figure 1. The total number of cardiac procedures was 16.2% smaller in 2020 compared to the previous year. The greatest proportional reduction was found for OVRP (19.4%) followed by PCI (16.1%), CABG (15.3%), and TVR (10.4%).

In Table 1, the variables for patients with COPD who underwent cardiac procedures in 2019 and 2020 are presented. The proportion of women rose significantly from 13.03% in 2019 to 14.88% in 2020 (*p* = 0.015). None of the clinical characteristics showed a significant change over time. The most prevalent chronic heart conditions were ischemic heart disease and valvular heart disease, which were recorded in around 85% and 35% of people with COPD, respectively. Diabetes was present in approximately 40%, and chronic kidney disease was present in 17%.

Regarding hospital outcomes, LOHS was significantly lower in 2020 than in 2019 (9.37 days vs. 10.13 days; *p* = 0.004) and crude IHM was significantly higher (5.32% vs. 4.33%; *p* = 0.035).

In Table 2, the demographic characteristics, clinical variables, and hospital outcomes of patients with COPD who underwent a coronary artery procedure (PCI or CABG) in Spain in 2019 and 2020 are presented. For both procedures there was a significant male predominance (around 90% men), and the mean age and CCI were higher for those receiving PCI compared to CABG.

Among those who underwent PCI, from 2019 to 2020, the only significant changes observed were in the proportion of women, which rose from 11.41% to 13.22% (*p* = 0.035); in mean CCI, which increased from 3.13 to 3.22 (*p* = 0.044); and in LOHS, which decreased from 7.56 days to 6.88 days (*p* = 0.002). IHM increased insignificantly, from 3.96% in 2019 to 4.72% in 2020 (*p* = 0.154).

The distribution according to study variables was very similar for those people with COPD who underwent a CABG in 2019 and 2020. However, the proportion of those admitted to the ICU increased from 76.03% to 82.01% (0.034). IHM rose slightly from 5.01% to 5.4% (*p* = 0.80) over time.

A description of the people with COPD who underwent a procedure affecting the heart valves—open replacement or TVR—is shown in Table 3. The proportion of men was around 80% for both procedures and no changes were observed from 2019 to 2020. Those who received a TVR were 9 years older than those who received an OVRP (79 years vs. 70 years) and had a higher mean CCI (2.96 vs. 2.50).

Among those who received an OVRP, the characteristics of those who were operated on in 2019 and 2010 are very similar. However, a significant increase can be observed in the proportion of patients requiring admission to the ICU, which increased from 75.66% in 2019 to 83.47% in 2020 (*p* = 0.002). IHM was higher in 2020 (8.78%) compared to the previous year (6.74%), but this difference was not statistically significant (*p* = 0.208).

For TVR, as seen in Table 3, the people with COPD who underwent this procedure did not differ significantly from one another according to study variables obtained for 2019 and 2020. The increase in IHM observed in 2020 with respect to 2019 was very small (4.26% vs. 3.56%; *p* = 0.622).

The results of the multivariable logistic regression models used to assess the differences in IHM from 2019 to 2020 after adjusting for age, sex, and CCI are shown in Table 4. For all of the procedures that were studied, the Odds Ratios (ORs) showed values over 1, suggesting a higher risk of dying in 2020 compared to 2019. However, the ORs were only statistically significant for the “any cardiac procedure” (1.18, 95% CI 1.03–1.47). This means that the risk of dying during hospitalization was 18% higher if people with COPD underwent these procedures during the year in which the pandemic began compared to the previous year.

As expected, a higher CCI increased IHM for each of the procedures analyzed (Table S2). Finally, after multivariable adjustment, the probability of dying in hospital was higher for women and older patients with COPD who underwent CABG or OVRP.

### Sensitivity Analysis

Table S2 presents IHM for cardiac procedures performed in the four years before the onset of the COVID-19 pandemic. For “*any procedure*”, a significant change was observed to have taken place over time. IHM ranged from 4.09% to 4.55% in the pre-pandemic years, increasing to 5.32% in 2020 (*p* = 0.003). For the rest of the procedures, the results for the period 2016–2020 were not significant, similar to what was observed in 2019–2020.

The clinical characteristics and hospital outcomes of patients with and without COVID-19 infection who underwent a cardiac procedure in 2020 are shown in Table S3. The total number of patients with a diagnosis of COVID-19 infection was 30, of which 22 (73.33%) underwent a PCI, 4 a CABG and an OVRP, and 2 a TVR. This distribution is very similar for those without COVID-19 infection. Patients with COVID-19 infection had a significantly higher mean CCI (4.27 vs. 3.08; *p* < 0.001), higher mean LOHS (16.6 days vs 9.31 days; *p* = 0.001), and IHM (36.67% vs 5.07%; *p* < 0.001).

When each patient without COVID-19 infection was matched by type of procedure, sex, age, and month of intervention with a patient with this infection hospitalized in 2019, we found the results shown in Table S4. After matching the differences in CCI, LOHS and IHM remained statistically significant, confirming worse clinical profiles and hospital outcomes in patients with COVID-19 infection when compared to patients hospitalized in 2019.

## 4. Discussion

Our finding clarified the impact of the COVID-19 pandemic on the performance of cardiac procedures in the COPD population. We observed that the total number of such interventions undertaken in COPD patients was 16.2% lower in 2020 compared to the previous year. The greatest proportional reduction was observed for OVRP (19.4%) followed by PCI (16.1%), CABG (15.3%), and TVR (10.4%). Similarly, we also found a higher risk of death in these patients in 2020 compared to in 2019. In fact, the risk of death during hospitalization was 18% higher in COPD patients who underwent these procedures during the pandemic year compared to in the previous year. Taken together, these findings have important clinical implications. They indicate that the COPD patients who underwent cardiac procedures during the pandemic might potentially have higher-acuity presentations or be diagnosed at a later point in their disease progression compared to patients before the COVID-19 pandemic, as other authors have already suggested [17]. However, we found no significant differences in the demographic variables, CCI, and clinical characteristics between 2019 and 2020. Therefore, it is not clear why increased mortality was observed in patients in 2020. In any case, this study may only serve to generate hypotheses about this specific topic. There are several factors that may explain the decline in cardiac procedures during the pandemic period. The initial interruption of patient referral pathways, such as reduced noninvasive outpatient testing for coronary artery disease during the pandemic, might be partly responsible [18]. It has also been documented that a decrease in acute coronary syndrome hospitalizations occurred, which was partly related to the refusal of patients to go to hospital for fear of contracting SARS-CoV-2. Another influencing factor is the perioperative dependence of cardiac surgery patients on intensive care units (ICUs), which supported a significant part of the response to the COVID-19 pandemic [8,19,20,21].

A greater reduction was observed in OVRP than in TVR in our study. Perek et al. [22] also found similar changes in cardiac procedures during the coronavirus pandemic with more percutaneous interventions being performed, whereas the open surgical approach was predominantly chosen for urgent patients requiring more complex procedures. Percutaneous techniques are minimally invasive and enable faster recovery, and many TVR cases, after careful post-procedure monitoring in the recovery room, may be transferred safely to the cardiology ward, reducing a need for ICU admissions [23].

The decline in the number of PCI procedures observed in our study is consistent with prior reports [7,24,25]. Possible explanations, besides patient reluctance to go to the hospital because of the fear of contracting COVID-19 infection as previously noted, include misdiagnosis and increased use of pharmacological reperfusion due to COVID-19 infection [26].

Importantly, we observed that there was an increase in mortality rates in COPD patients who underwent cardiac procedures during the COVID-19 pandemic. Inconclusive results have been reported by other studies that have observed either increases or no changes in IHM in relation to such interventions before and during the COVID-19 pandemic [27,28,29,30,31]. In a recent systematic review and meta-analysis, it was reported that COVID-19 patients undergoing cardiac surgery have poor outcomes [32]. These results were confirmed in our investigation where IHM was much higher among patients with COVID-19 infection (Tables S3 and S4). However, in our population, only 30 COPD patients with COVID-19 underwent the studied cardiac procedures. The reason for this small number is because, unless the need for the cardiac procedure was very urgent and impossible to postpone, the intervention was delayed until the patient was no longer infected with COVID-19 and the patient was not infectious. In any case, we cannot exclude the influence of SARS-CoV-2. Various studies have indicated that SARS-CoV-2 infection acts on endothelial cells not only in the lungs but also in blood vessels and the heart, causing deterioration processes, such as inflammation and oxidative stress, and numerous cardiovascular complications. In addition, new data shows that even asymptomatic patients, not just patients with symptoms, can experience these complications immediately after illness, in the next 30 days, and even after a year [32,33].

An interesting finding in our study was that the probability of dying in hospital was higher for COPD women who underwent CABG or OVRP. In the same way, Dixon et al. [34] conducted a systematic review and meta-analysis and concluded that females are at a greater risk of short-term mortality and post-operative stroke than males following CABG and valve surgery combined with CABG. Our findings add to the existing published literature on gender disparities after cardiac surgery by providing a description of IHM after cardiac procedures in COPD patients.

Several practical implications can be derived from our investigation. It is necessary for health services to prepare or update protocols that prioritize cases to be operated on and therapeutic alternatives to surgery for patients who are at a lower risk. It is also important to optimize health organizations so that patients can be discharged as soon as possible to reduce their risk of being infected and to ensure free beds for new patients. Furthermore, coordination improvements are required at the national or regional level so that all health resources can be used efficiently. Solutions include considering the creation of centers that are exclusively dedicated to the treatment of positive or disease-free patients [35,36,37].

In our country, other authors have proposed new programs, including conducting surgery in the afternoon and increasing surgical activity in the summer months [35,36,37].

## 5. Limitations

This study must be interpreted in the context of its limitations. First, our data were obtained from an administrative database containing information recorded by physicians in discharge reports; therefore, data on medical treatments, the duration and severity of COPD or heart disease, hemodynamic and echocardiographic parameters, and preoperative risk metrics have not been recorded. Second, as we analyzed relatively low-risk procedures in this study, caution is needed when extrapolating our conclusions to higher-risk cardiac procedures or to other surgical disciplines. Third, the low risk of the procedures studied meant that the IHM comparison was conducted with a small sample size. However, the sensitivity analysis that included three more years (2016, 2017, and 2018) confirmed the results obtained for the years 2019 and 2020. Fourth, we do not have post-discharge follow-up data on mortality. Fifth, our database does not include information on the reason why a surgery was not conducted (patient refusal or postponement or cancellation by health services). However, studies conducted in Spain have concluded that most surgeries were postponed or cancelled by health authorities due to hospitals and intensive care units being overloaded with COVID-19 infected patients [34,35,36]. Finally, the codes to identify the study outcomes were inferred from ICD-10, so the existence of misclassification bias cannot be discarded. In addition, there may have been changes in coding practices as a consequence of the pandemic. Future work should be undertaken to understand the effects of the pandemic on postprocedural long-term outcomes.

## 6. Conclusions

In conclusion, our national evaluation demonstrates a 16.2% decrease in cardiac procedures during the COVID-19 pandemic in relation to the preceding period. On the other hand, IHM was higher in 2020 than it was in 2019. Suffering a COVID-19 infection was associated with significantly higher mortality after cardiac procedures. These findings highlight the importance of early recognition of COPD patients with cardiovascular diseases requiring a cardiac procedure.

## Figures and Tables

**Table 1:** Demographic and clinical characteristics and in-hospital outcomes of patients with chronic obstructive pulmonary disease who underwent “any cardiac procedure” in Spain in 2019 and 2020. Analysis of the Spanish National Hospital Discharge Database.

Variables	Year 2019	Year 2020	*p*-Value
N	4483	3757	NA
Men, n (%)	3899 (86.97)	3198 (85.12)	0.015
Women, n (%)	584 (13.03)	559 (14.88)	
Age, mean (SD)	71.81 (9.43)	71.69 (9.47)	0.582
40–54 years, n (%)	183 (4.08)	170 (4.52)	0.626
55–64 years, n (%)	834 (18.6)	688 (18.31)	
65–74 years, n (%)	1588 (35.42)	1359 (36.17)	
≥75 years, n (%)	1878 (41.89)	1540 (40.99)	
CCI index, mean (SD)	3.03 (1.64)	3.09 (1.70)	0.098
Diabetes, n (%)	1793 (40)	1517 (40.38)	0.724
Asthma, n (%)	115 (2.57)	106 (2.82)	0.473
Stroke, n (%)	48 (1.07)	39 (1.04)	0.885
Peripheral vascular disease, n (%)	605 (13.50)	509 (13.55)	0.945
Ischemic heart disease, n (%)	3783 (84.39)	3205 (85.31)	0.246
Valvular heart disease, n (%)	1558 (34.75)	1347 (35.85)	0.298
Atrial fibrillation, n (%)	1095 (24.43)	933 (24.83)	0.668
Heart failure, n (%)	1161 (25.9)	982 (26.14)	0.805
Chronic kidney disease, n (%)	766 (17.09)	624 (16.61)	0.564
COVID-19, n (%)	NA	30 (0.8)	-
Oxygen prior to hospital admission, n (%)	195 (4.35)	152 (4.05)	0.494
Non-invasive mechanical ventilation, n (%)	151 (3.37)	135 (3.59)	0.578
Invasive mechanical ventilation, n (%)	319 (7.12)	238 (6.33)	0.160
Dialysis, n (%)	121 (2.7)	76 (2.02)	0.045
Admission to ICU, n (%)	2027 (45.22)	1733 (46.13)	0.408
LOHS, mean (SD)	10.13 (9.42)	9.37 (9.16)	0.004
IHM, n (%)	194 (4.33)	200 (5.32)	0.035

“Any cardiac procedure” included: percutaneous coronary intervention; coronary artery bypass graft; open valve replacement procedures for aortic, mitral, pulmonary, and tricuspid valves; and transcatheter valve replacement for aortic, mitral, pulmonary, and tricuspid valves (ICD10 codes for these procedures are shown in Supplementary Table S1). CCI: Charlson comorbidity index; ICU: intensive care unit; LOHS: length of hospital stay; IHM: in-hospital mortality; NA: not available as this ICD10 code was created in 2020.

**Table 2:** Demographic and clinical characteristics and in-hospital outcomes of patients with chronic obstructive pulmonary disease who underwent a Percutaneous Coronary Intervention or a coronary artery bypass graft in Spain in 2019 and 2020. Analysis of the Spanish National Hospital Discharge Database.

	Percutaneous Coronary Intervention			Coronary Artery Bypass Graft		
Variable	Year 2019	Year 2020	*p*-Value	Year 2019	Year 2020	*p*-Value
N (%)	3182	2670	NA	459 (100)	389 (100)	NA
Men, n (%)	2819 (88.59)	2317 (86.78)	0.035	422 (91.94)	347 (89.2)	0.172
Women, n (%)	363 (11.41)	353 (13.22)		37 (8.06)	42 (10.8)	
Age, mean (SD)	71.51 (9.62)	71.4 (9.75)	0.644	69.6 (7.66)	69.03 (7.25)	0.270
40–54 years, n (%)	137 (4.31)	138 (5.17)	0.469	19 (4.14)	11 (2.83)	0.105
55–64 years, n (%)	641 (20.14)	524 (19.63)		93 (20.26)	86 (22.11)	
65–74 years, n (%)	1116 (35.07)	936 (35.06)		210 (45.75)	201 (51.67)	
≥75 years, n (%)	1288 (40.48)	1072 (40.15)		137 (29.85)	91 (23.39)	
CCI index, mean (SD)	3.13 (1.65)	3.22 (1.71)	0.044	2.99 (1.54)	2.99 (1.66)	0.990
Diabetes, n (%)	1315 (41.33)	1088 (40.75)	0.655	200 (43.57)	194 (49.87)	0.067
Asthma, n (%)	77 (2.42)	76 (2.85)	0.308	10 (2.18)	8 (2.06)	0.902
Stroke, n (%)	25 (0.79)	24 (0.9)	0.636	6 (1.31)	3 (0.77)	0.448
Peripheral vascular disease, n (%)	372 (11.69)	318 (11.91)	0.796	87 (18.95)	74 (19.02)	0.980
Valvular heart disease, n (%)	545 (17.13)	508 (19.03)	0.060	197 (42.92)	159 (40.87)	0.548
Atrial fibrillation, n (%)	577 (18.13)	505 (18.91)	0.444	136 (29.63)	111 (28.53)	0.727
Heart failure, n (%)	802 (25.2)	672 (25.17)	0.975	108 (23.53)	102 (26.22)	0.366
Chronic kidney disease, n (%)	530 (16.66)	439 (16.44)	0.826	74 (16.12)	55 (14.14)	0.423
COVID-19, n (%)	NA	22 (0.82)	-	NA	4 (1.03)	-
Oxygen prior to hospital admission, n (%)	141 (4.43)	117 (4.38)	0.927	4 (0.87)	6 (1.54)	0.367
Non-invasive mechanical ventilation, n (%)	93 (2.92)	80 (3)	0.869	29 (6.32)	27 (6.94)	0.716
Invasive mechanical ventilation, n (%)	114 (3.58)	95 (3.56)	0.960	83 (18.08)	57 (14.65)	0.180
Dialysis, n (%)	57 (1.79)	40 (1.5)	0.382	30 (6.54)	21 (5.40)	0.201
Admission to ICU, n (%)	1143 (35.92)	959 (35.92)	0.998	349 (76.03)	319 (82.01)	0.034
LOHS, mean (SD)	7.56 (7.48)	6.88 (6.7)	0.002	19.55 (19.44)	17.62 (15.6)	0.115
IHM, n (%)	126 (3.96)	126 (4.72)	0.154	23 (5.01)	21 (5.4)	0.800

CCI: Charlson comorbidity index; ICU: intensive care unit; LOHS: length of hospital stay; IHM: in-hospital mortality; NA: not available as this ICD10 code was created in 2020.

**Table 3:** Demographic and clinical characteristics and in-hospital outcomes of patients with chronic obstructive pulmonary disease who underwent open-heart valve replacement or a transcatheter valve implantation in Spain in 2019 and 2020. Analysis of the Spanish National Hospital Discharge Database.

	Open Heart Valve Replacement			Transcatheter Valve Replacement		
Variable	Year 2019	Year 2020	*p*-Value	Year 2019	Year 2020	*p*-Value
N (%)	608 (100)	490 (100)	NA	393	352	NA
Men, n (%)	487 (80.1)	377 (76.94)	0.204	315 (80.15)	278 (78.98)	0.691
Women, n (%)	121 (19.9)	113 (23.06)		78 (19.85)	74 (21.02)	
Age, mean (SD)	70.47 (8.22)	70.08 (7.69)	0.416	78.89 (7.38)	79.53 (6.57)	0.217
40–54 years, n (%)	26 (4.28)	20 (4.08)	0.257	2 (0.51)	1 (0.28)	0.471
55–64 years, n (%)	113 (18.59)	90 (18.37)		15 (3.82)	7 (1.99)	
65–74 years, n (%)	248 (40.79)	227 (46.33)		78 (19.85)	68 (19.32)	
≥75 years, n (%)	221 (36.35)	153 (31.22)		298 (75.83)	276 (78.41)	
CCI index, mean (SD)	2.50 (1.45)	2.49 (1.47)	0.825	2.97 (1.68)	2.95 (1.7)	0.595
Diabetes, n (%)	191 (31.41)	153 (31.22)	0.946	146 (37.15)	145 (41.19)	0.259
Asthma, n (%)	22 (3.62)	9 (1.84)	0.076	11 (2.8)	15 (4.26)	0.278
Stroke, n (%)	12 (1.97)	9 (1.84)	0.869	7 (1.78)	4 (1.14)	0.466
Peripheral vascular disease, n (%)	106 (17.43)	78 (15.92)	0.504	67 (17.05)	60 (17.05)	0.999
Ischemic heart disease, n (%)	206 (33.88)	178 (36.33)	0.398	132 (33.59)	136 (38.64)	0.152
Atrial fibrillation, n (%)	276 (45.39)	228 (46.53)	0.707	171 (43.51)	142 (40.34)	0.381
Heart failure, n (%)	155 (25.49)	138 (28.16)	0.320	141 (35.88)	122 (34.66)	0.728
Chronic kidney disease, n (%)	86 (14.14)	69 (14.08)	0.976	103 (26.21)	83 (23.58)	0.408
COVID-19, n (%)	NA	4 (0.82)	-	NA	2 (0.57)	-
Oxygen prior to hospital admission, n (%)	10 (1.64)	7 (1.43)	0.773	40 (10.18)	25 (7.1)	0.137
Non-invasive mechanical ventilation, n (%)	28 (4.61)	32 (6.53)	0.163	14 (3.56)	6 (1.7)	0.117
Invasive mechanical ventilation, n (%)	133 (21.88)	107 (21.84)	0.988	16 (4.07)	7 (1.99)	0.101
Dialysis, n (%)	37 (6.09)	24 (4.9)	0.393	9 (2.29)	12 (3.41)	0.357
Admission to ICU, n (%)	460 (75.66)	409 (83.47)	0.002	198 (50.38)	163 (46.31)	0.267
LOHS, mean (SD)	18.52 (18.32)	18.7 (18.07)	0.873	11.36 (10.25)	10.03 (8.7)	0.058
IHM, n (%)	41 (6.74)	43 (8.78)	0.208	14 (3.56)	15 (4.26)	0.622

CCI: Charlson comorbidity index; ICU: intensive care unit; LOHS: length of hospital stay; IHM: in-hospital mortality; NA: not available as this ICD10 code was created in 2020.

**Table 4:** Multivariable logistic regression models to assess the change from 2019 to 2020 and the variables associated with in-hospital mortality among people with chronic obstructive pulmonary disease who underwent any cardiac procedure, percutaneous coronary intervention (PCI), coronary artery bypass graft (CABG), open valve replacement procedures (OVRP), and transcatheter valve replacement (TVR).

	Any Cardiac Procedure	PCI	CABG	OVRP	TVR
VARIABLE	OR (95% CI)	OR(95% CI)	OR(95% CI)	OR(95% CI)	OR(95% CI)
Men	Reference	Reference	Reference	Reference	Reference
Women	1.78 (1.37–2.32)	1.23 (0.84–1.81)	2.99 (1.30–6.87)	3.34 (2.07–5.38)	0.86 (0.32–2.31)
CCI *	1.28 (1.22–1.35)	1.28 (1.21–1.36)	1.40 (1.15–1.60)	1.39 (1.22–1.59)	1.25 (1.03–1.52)
Age *	1.02 (1.00–1.04)	1.01 (0.96–1.06)	1.05 (1.02–1.08)	1.04 (1.01–1.07)	1.08 (0.95–1.21)
Year 2019	Reference	Reference	Reference	Reference	Reference
Year 2020	1.18 (1.03–1.47)	1.16 (0.90–1.50)	1.03 (0.54–1.90)	1.29 (0.81–2.03)	1.19 (0.57–2.53)

The variables included in the final model are shown in the table. OR: Odds Ratio. 95% CI: 95% confidence interval. CCI; Charlson comorbidity index. Reference: reference category for that variable. * Age and CCI were introduced in the models as continuous variables.

## Data Availability

According to the contract signed with the Spanish Ministry of Health and Social Services, which provided access to the databases from the Spanish National Hospital Database (RAE-CMBD, Registro de Actividad de Atención Especializada. Conjunto Mínimo Básico de Datos, Registry of Specialized Health Care Activities. Minimum Basic Data Set), we cannot share the databases with any other investigator, and we have to destroy the databases once the investigation has concluded. Consequently, we cannot upload the databases to any public repository. However, any investigator can apply for access to the databases by filling out the questionnaire available at: http://www.msssi.gob.es/estadEstudios/estadisticas/estadisticas/estMinisterio/SolicitudCMBDdocs/Formulario_Peticion_Datos_CMBD.pdf (accessed on 13 March 2022). All other relevant data are included in the paper.

## Supplementary Materials

The following supporting information can be downloaded at: https://www.mdpi.com/article/10.3390/jcm11133924/s1. Table S1: International Classification of Disease, 10th edition, (ICD-10) codes for the clinical diagnoses and procedures used in this investigation. Table S2: In-hospital mortality of patients with chronic obstructive pulmonary disease who underwent a percutaneous coronary intervention, coronary artery bypass graft, open surgical valve replacement, transcatheter valve replacement or any of these procedures from 2016 to 2020 in Spain. Analysis of the Spanish National Hospital Discharge Database. Table S3: Demographic and clinical characteristics and in-hospital outcomes of patients with chronic obstructive pulmonary disease who underwent a cardiac procedure in Spain in 2020 according to the presence of infection with COVID-19. Analysis of the Spanish National Hospital Discharge Database. Table S4: Demographic and clinical characteristics and in-hospital outcomes of patients with chronic obstructive pulmonary disease who underwent a cardiac procedure in Spain in 2020 in patients with COVID-19 infection and matched patients without COVID-19 who underwent a cardiac procedure in Spain in 2019. Analysis of the Spanish National Hospital Discharge Database.
