# Supplementary material for: Impact of the COVID-19 Pandemic on the Use and Outcomes of Cardiac Procedures in COPD Patients
Source: J Clin Med. 2022 Jul 5;11(13):3924. doi: 10.3390/jcm11133924 (PMC9267656; doi:10.3390/jcm11133924)
Supplement: Supplementary file 1 [file jcm-11-03924-s001.zip › jcm-1705630-supplementary.pdf]

Table S1. International Classification of Disease, 10th edition, (ICD-10) codes for the clinical diagnoses and procedures used in this investigation.

Procedures	ICD-10 codes
Percutaneous coronary intervention.	02703XX, 02704xx, 02713XX, 02714xx, 02723XX, 02724xx, 02733XX, 02734xx
Coronary artery bypass graft	02100XX, 02110XX, 02120XX, 02130XX
Open heart valve replacement	02RF0XX, 02RG0XX, 02RH0XX, 02RJ0XX
Trans-catheter valve implantation	02RF3xx; 02RG3xx; 02RH3xx; 02RJ3xx
Oxygen prior to hospital admission	Z99.81
Non-invasive mechanical ventilation	5A09357, 5A09457, 5A09557
Invasive mechanical ventilation	5A1945Z, 5A1955Z, 5A1935Z
Dialysis	5A1D xxx
**Diagnosis**	**ICD-10 codes**
Diabetes	E10.xxx ,E11.xxx
Asthma	J45
Stroke	I60-I63
Peripheral vascular disease	I70.x, I71.x, I73.1, I73.8, I73.9, I77.1, I79.0, I79.2, K55.1, K55.8, K55.9, Z95.8, Z95.9
Ischemic heart disease	I20-I25
Valvular heart disease	I05-I08, I33-I39
Atrial fibrillation	I48.0, I48.1, I48.2, I48.91
Heart failure	I09.9, I11.0, I13.0, I13.2, I25.5, I42.0, I42.5–I42.9, I43.x, I50.x, P29.0
Chronic kidney disease	N18
COVID 19	B34.2, B97.29, U07.1

Table S2. In hospital mortality of patients with Chronic Obstructive Pulmonary Disease who underwent a percutaneous coronary intervention, coronary artery bypass graft, open surgical valve replacement, transcatheter valve implantation or any of these procedures from 2016 to 2020 in Spain. Analysis of the Spanish National Hospital Discharge Database.

	**2016**	**2017**	**2018**	**2019**	**2020**	***p* for trend**
Percutaneous coronary intervention.	111(4.01)	125(4.26)	120(3.97)	126(3.96)	126(4.72)	0.392
Coronary artery bypass graft	19(4.94)	22(4.25)	15(3.5)	23(5.01)	21(5.4)	0.084
Open heart valve replacement	42(6.72)	44(6.54)	39(6.7)	41(6.74)	43(8.78)	0.389
Trans-catheter valve implantation	10(4.85)	12(4.24)	12(3.89)	14(3.56)	15(4.26)	0.429
Any procedure	176(4.55)	186(4.17)	184(4.09)	194(4.33)	200(5.32)	0.003

Table S3. Demographic and clinical characteristics and in-hospital outcomes of patients with chronic obstructive pulmonary disease who underwent cardiac procedure in Spain in 2020 according to the presence of infection with COVID-19. Analysis of the Spanish National Hospital Discharge Database.

**Variables**	**No COVID 19 infection**	**COVID-19 infection**	***p*-value**
N	3727(100)	30(100)	NA
PCI n (%)	2648(71.05)	22(73.33)	0.591
CABG. n (%)	385(10.33)	4(13.33)	0.783
OVRP. n (%)	486(13.04)	4(13.33)	0.610
TVI. n (%)	350(9.39)	2(6.67)	0.962
Men n (%)	3171(85.08)	27(90)	
Women n (%)	556(14.92)	3(10)	0.451
Age. mean (SD)	71.68(9.46)	73(10)	0.449
CCI index. mean (SD)	3.08(1.7)	4.27(1.98)	<0.001
Diabetes, n (%)	1501(40.27)	16(53.33)	0.146
Asthma n (%),n (%)	105(2.82)	1(3.33)	0.865
Stroke n (%)	39(1.05)	0(0)	0.573
Peripheral vascular disease n (%)	507(13.6)	2(6.67)	0.269
Ischemic heart disease n (%)	3178(85.27)	27(90)	0.466
Valvular heart disease n (%)	1336(35.85)	11(36.67)	0.926
Atrial fibrillation n (%)	922(24.74)	11(36.67)	0.132
Heart failure n (%)	968(25.97)	14(46.67)	0.010
Chronic kidney disease n (%)	616(16.53)	8(26.67)	0.137
Oxygen prior to hospital admission, n (%)	151(4.05)	1(3.33)	0.842
Non-invasive mechanical ventilation. n (%)	131(3.51)	4(13.33)	0.004
Invasive mechanical ventilation. n (%)	234(6.28)	4(13.33)	0.114
Dialysis. n (%)	74(1.99)	2(6.67)	0.070
Admission to ICU. n (%)	1719(46.12)	14(46.67)	0.953
LOHS, mean (SD)	9.31(8.68)	16.6(13.48)	0.001
IHM. n (%)	189(5.07)	11(36.67)	<0.001

Table S4. Demographic and clinical characteristics and in-hospital outcomes of patients with chronic obstructive pulmonary disease who underwent cardiac procedure in Spain in 2020 in patients with COVID-19 infection and matched patients without COVID-19 who underwent cardiac procedure in Spain in 2019. Analysis of the Spanish National Hospital Discharge Database.

**Variables**	**Matched patients without COVID-19 infection ***	**COVID-19 infection**	***p*-value**
N	30(100)	30(100)	NA
PCI n (%)	22(73.33)	22(73.33)	NA
CABG. n (%)	4(13.33)	4(13.33)	NA
OVRP. n (%)	4(13.33)	4(13.33)	NA
TVI. n (%)	2(6.67)	2(6.67)	NA
Men n (%)	27(90)	27(90)	
Women n (%)	3(10)	3(10)	NA
Age. mean (SD)	73(10)	73(10)	NA
CCI index. mean (SD)	2.9(1.45)	4.27(1.98)	0.003
Diabetes, n (%)	13(43.33)	16(53.33)	0.438
Asthma n (%),n (%)	1(3.33)	1(3.33)	0.999
Stroke n (%)	1(3.33)	0(0)	NA
Peripheral vascular disease n (%)	5(16.67)	2(6.67)	0.228
Ischemic heart disease n (%)	26(86.67)	27(90)	0.688
Valvular heart disease n (%)	9(30)	11(36.67)	0.584
Atrial fibrillation n (%)	8(26.67)	11(36.67)	0.405
Heart failure n (%)	5(16.67)	14(46.67)	0.012
Chronic kidney disease n (%)	2(6.67)	8(26.67)	0.038
Oxygen prior to hospital admission, n (%)	0(0)	1(3.33)	NA
Non-invasive mechanical ventilation. n (%)	0(0)	4(13.33)	NA
Invasive mechanical ventilation. n (%)	1(3.33)	4(13.33)	0.161
Dialysis. n (%)	0(0)	2(6.67)	0.150
Admission to ICU. n (%)	14(46.67)	14(46.67)	0.999
LOHS, mean (SD)	7.8(7.34)	16.6(13.48)	0.006
IHM. n (%)	1(3.33)	11(36.67)	0.001

* Each patient with COVID-19 infection was matched with a patient without COVID-19 infection who underwent the same procedure with identical age, sex and month of intervention in year 2019.
